# Net water uptake and lesion extent as joint expressions of ischaemic injury: a comment on the TENSION secondary analysis

**DOI:** 10.1093/esj/aakag095

**Published:** 2026-08-18

**Authors:** Apostolos Safouris, Wim H van Zwam

**Affiliations:** Stroke Unit, Metropolitan Hospital, Piraeus, Greece; Department of Radiology and Nuclear Medicine, Maastricht University Medical Center, Maastricht, The Netherlands

**Keywords:** cerebral oedema, mechanical thrombectomy, mediation analysis

Dear Editor,

We read with great interest the secondary analysis of TENSION by Broocks and colleagues, which adds an important mechanistic dimension to the large-core thrombectomy literature.^1^ The demonstration that thrombectomy reduces oedema progression, and that this reduction mediates a substantial part of the functional benefit, is both elegant and clinically resonant.

Net water uptake and lesion extent are, in our reading, 2 expressions of a single underlying process: the severity and the extent of ischaemic injury. As the authors’ own work has established, net water uptake is a measure of lesion water concentration, so that net water uptake multiplied by lesion volume yields the absolute volume of ischaemic water uptake: oedema as a quantity rather than a density.^2^ This raises a question about the form of the mediator. Rather than entering ΔNWU (net water uptake) and ΔASPECTS (Alberta Stroke Program Early CT Score) as 2 separate variables, a mediation model built on their product (total ischaemic water uptake) might be the more complete marker of overall injury and would test directly whether outcome depends on severity and extent multiplicatively rather than through 2 additive channels. Such a composite might plausibly explain a larger share of the treatment effect than the 2 considered separately.

As a point about the present analysis rather than the choice of marker, we would add 1 limitation on the comparison of the 2 reported mediated proportions (34% for ΔNWU, 14% for ΔASPECTS). These derive from 2 single-mediator models estimated on different patient sets (all patients with available ASPECTS in one, and the subset in whom net water uptake could be quantified in the other) and are therefore referenced to somewhat different total effects, which may temper the direct comparability of the 2 fractions. Because the 2 mediators are correlated, each single-mediator estimate will to some degree reflect the other pathway; estimating both jointly against a common total effect^3^ would make the oedema-specific contribution easier to interpret. We appreciate that any such decomposition carries the usual cautions about post-treatment mediators, which the authors rightly acknowledge, and that modelling oedema together with infarct extent against a common total effect is one route to doing so, as Sarraj and colleagues did for reperfusion benefit.^4^

## Data Availability

Data sharing is not applicable, as no new data were generated or analysed for this correspondence.
